# A New Initialization Approach in Particle Swarm Optimization for Global Optimization Problems

**DOI:** 10.1155/2021/6628889

**Published:** 2021-05-17

**Authors:** Waqas Haider Bangyal, Abdul Hameed, Wael Alosaimi, Hashem Alyami

**Affiliations:** ^1^Dept. of Computer Science, University of Gujrat, Gujrat, Pakistan; ^2^Dept. of Computer Science, Iqra University, Islamabad, Pakistan; ^3^Department of Information Technology, College of Computers and Information Technology, Taif University, Taif, Saudi Arabia; ^4^Department of Computer Science, College of Computers and Information Technology, Taif University, Taif, Saudi Arabia

## Abstract

Particle swarm optimization (PSO) algorithm is a population-based intelligent stochastic search technique used to search for food with the intrinsic manner of bee swarming. PSO is widely used to solve the diverse problems of optimization. Initialization of population is a critical factor in the PSO algorithm, which considerably influences the diversity and convergence during the process of PSO. Quasirandom sequences are useful for initializing the population to improve the diversity and convergence, rather than applying the random distribution for initialization. The performance of PSO is expanded in this paper to make it appropriate for the optimization problem by introducing a new initialization technique named WELL with the help of low-discrepancy sequence. To solve the optimization problems in large-dimensional search spaces, the proposed solution is termed as WE-PSO. The suggested solution has been verified on fifteen well-known unimodal and multimodal benchmark test problems extensively used in the literature, Moreover, the performance of WE-PSO is compared with the standard PSO and two other initialization approaches Sobol-based PSO (SO-PSO) and Halton-based PSO (H-PSO). The findings indicate that WE-PSO is better than the standard multimodal problem-solving techniques. The results validate the efficacy and effectiveness of our approach. In comparison, the proposed approach is used for artificial neural network (ANN) learning and contrasted to the standard backpropagation algorithm, standard PSO, H-PSO, and SO-PSO, respectively. The results of our technique has a higher accuracy score and outperforms traditional methods. Also, the outcome of our work presents an insight on how the proposed initialization technique has a high effect on the quality of cost function, integration, and diversity aspects.

## 1. Introduction

Optimization is considered the most productive field of research for many decades. Advanced optimization algorithms are required, as the problems of the real world evolve time towards complexity. The key purpose is to obtain the fitness function's optimum value [1]. The classification is an attempt to identify groups of certain categories of data. Moreover, the training data have many features that play a significant role in segregating the knowledge according to the classes' prearranged categories. Globally, a massive growth is recognized in various data classification applications, such as organic compound analysis, television audience share prediction, automatic abstraction, credit card fraud detection, financial projection, targeted marketing, and medical diagnosis [2]. In evolutionary computation, data classification builds its model based on the genetic process and natural evolution [3]. These techniques are adaptive and robust, which perform global exploration instead of candidate solutions for the extraction of information on large datasets.

The fundamental domain of artificial intelligence is swarm intelligence (SI), which discusses the developmental methods that govern the multiagent mechanism by systemic architecture and are influenced by the behaviour of social insects such as ants, wasps, bees, and termites. They are also encouraged by other social animal colonies, such as bird flocking or fish schooling [4]. In the research of cellular robotic systems, first, the word SI is defined by Beni and Wang [5]. Researchers have been associated with social insect communities for decades, but for a long time, researchers have not established the composition of their collective behaviour. Moreover, the society's autonomous agent is preserved as a nonsophisticated single, as it can deal with complicated issues. Complex tasks are accomplished effectively through an association with the single members of society as it strengthens the capacity to perform actions. In the field of optimization, different techniques of swarm intelligence are used.

Particle swarm optimization (PSO) is considered the most efficient population-based stochastic algorithm, suggested by Kennedy and Eberhart in 1995 [6], employed to deal with the global optimization problems. It has become the most successful technique to solve the optimization problems listed in the diversified domain of engineering due to simplicity and effectiveness. PSO includes the increment of the population in the candidate solution known as the swarm, which is investigating the new search spaces to aggregate the transformation of “flock of birds” while seeking the food. The communication of the information among all individuals is known as particles and all individuals lodged with findings of the rest of the swarm. Each individual follows the two essential rules for seeking: to return its old best point and ensure the best location of its swarm. With the advent of PSO, new methods were also encouraged to face the global problems with optimization in terms of solutions for fuzzy systems, artificial neural networks (ANNs) design, and evolutionary computing. ANNs' design [7] and function minimizations [8] are the most promising applications of evolutionary computing for solving complex optimization problems. PSO and evolutionary algorithms (EAs) have been efficiently used to measure the learning parameters, weight factors, and design of artificial neural networks [9, 10].

In the field of swarm evolutionary computing, the performance of PSO and other EAs are affected by the generation of random numbers during the initialization of the population into the multidimensional search space. PSO tends to achieve maximum performance when executed in the low dimensional search space. Therefore, the performance is expected to be low when the dimensionality of the problem is too high, which causes the particles to stick in the local solution [1, 11, 12]. Perseverance of the aforesaid behaviour becomes intolerable for a variety of real-life applications that contain a lot of local and global minima. Immature performance explains the reason for an inadequate population distribution of the swarm. It often implies that optimum solutions are more difficult to find if the particles do not accurately cover the entire search space, which could omit the global optimum [13–15]. This issue can be resolved by introducing a well-organized random distribution to initialize the swarm. These distributions can vary in structural design depending upon the family. Examples include pseudorandom sequences, probability sequences, and quasirandom sequences.

One of the classical ways of generating random numbers is by an inbuilt library (implemented in most programming languages, e.g., C or C++). The numbers are allocated uniformly by this inbuilt library. Research has proved that this technique is not useful for the uniform generation of random numbers and does not appear to obtain the lowest discrepancy [16]. Also, pseudorandom sequences of normal distributions reported better results compared to randomly distributed sequences [17]. Based on the design of the problem, the output of probability sequences, quasirandom sequences, and pseudorandom sequences varies. Due to variance in the generation of random numbers, pseudorandom sequences are better than quasirandom sequences for globally optimal solutions.

At this point, after a brief analysis of genetic algorithms, evolutionary algorithms, and PSO, we can infer that there is an insufficient amount of research has been performed to implement the pseudorandom sequences for population initialization. Despite this fact, to initialize the particles in the search space, we have proposed a novel pseudorandom initialization strategy called the WELL generator translated as (Well Equi-distributed Long-period Linear). We have compared the novel techniques with the basic random distribution and low-discrepancy sequence families, such as Sobol and Halton sequences on several complex unimodal and multimodal benchmark functions. The experimental findings have shown that WELL-based PSO initialization (WE-PSO) exceeds the other traditional PSO, PSO with Sobol-based initialization (SO-PSO), and PSO with Halton-based initialization (H-PSO) algorithms. Moreover, we have conducted the ANN training on real-world classification problems with quasirandom sequences. To compare the classifier's output, nine datasets were taken from the famous UCI repository. The results demonstrate that WE-PSO offered better results on real-world dynamic classification problems compared to PSO, SO-PSO, and H-PSO, respectively.

The remainder of the paper is structured as follows: in Section 2, related analysis is discussed. A general overview of the artificial neural network is found in Section 3.  In Section 4, the standard PSO is packed. The proposed technique is described in Section 5.  In Section 6, the findings are explained. Discussion, conclusion, and potential work are described in Section 7.

## 2. Related Work

### 2.1. Modified Initialization Approaches

Researchers have adopted various random number generators, i.e., pseudorandom, quasirandom, and probability sequences, to refine the efficiency of population-based evolutionary algorithms. The concept of using random number generator to initialize a swarm into multidimensional search space is not new. A comparison of low-discrepancy sequences with simple uniform distribution was carried out by the authors in [18] to assign the initial positions to particles in the search region. The study in [18] covers only the role of benchmark minimization function to verify the performance of different low-discrepancy sequence versions. Similarly, Kimura and Matsumura [19] optimized a genetic algorithm using the improved PSO variant to initialize the swarm based on the Halton sequence. The Halton series is under the umbrella of low-discrepancy sequences. The authors of [20] generated the comprehensive compression of Faure, Sobol, and Halton sequences, and after evaluation of the competitive outcomes, they declared a Sobol sequences as winner among others.

Van der Corput sequence associated with the quasirandom family was first carried out in [21]. For the initial parameters *d* = 1 and *b* = 2, the van der Corput sequences were generated, where *d* represents the problem dimensions and *b* is the base. The experimental results showed that for the difficult multidimensional optimization problems, the van der Corput sequence-based PSO outperforms the other quasirandom sequences, such as Faure sequence, Sobol sequence, and Halton sequence, respectively. Although, Halton-based PSO and Faure-based PSO gave better performance, when the optimization problem was low in dimensionality. Moreover, many researchers used the probability distribution to tune the different parameters of evolutionary algorithms. The family of probability sequences falls under the Gaussian distribution, Cauchy distribution, beta distribution, and exponential distribution, respectively. The authors in [22] tuned the PSO parameters using random sequences followed the use of an exponential distribution. Also, a detailed comparison of probability distributions is present in [23]. The experimental results revealed that the PSO based on exponential distribution performed well compared to the PSO based on Gaussian distribution and PSO based on beta distribution.

Similarly, the researchers applied a torus distribution [24] to initialize the improved Bat algorithm (I-BA). Torus-based initialization enhanced the diversity of swarm and showed better performance. In [2], the readers can find the source for applying several variations of probabilistic, quasirandom, and the uniform distribution in BA.

There are also other independent statistical methods to produce random numbers, apart from the probability distribution, pseudorandom distribution, and quasirandom distribution, used by various researchers to select an initial location of particles in multidimensional search space. The nonlinear simplex method (NSM) is an initialization method proposed by Parsopoulos and Vrahatis in [25]. The initialization based on centroidal Voronoi tessellations (CVTs) was suggested by Richards Ventura in [26]. The search region is divided into several blocks for the CVT process. In the first division of blocks, each particle gets a spot. The remaining particles, which have not been allocated a block yet, are further separated into subblocks. To allocate a block to a particle every time, the CVT generator used different permutations. The distance function is determined to disperse particles into blocks, and the less distant particles first reserve the entire block in the swarm. The initialization approach based on the CVT method is compared with the simple random distribution and the numerical results illustrated that PSO based on CVT was much better for the initialization of population.

A new technique called opposition-based initialization (O-PSO), inspired by opposition-based learning particles, was suggested by the authors in [27]. Certain particles took their positions in the opposite direction of search space, and O-PSO contributed to increasing the probability of having a global optimum at the beginning. To discover the search field in the opposite direction, which was parallel to the same direction, O-PSO enhanced the diversity of particles. Since good behaviour and poor behaviour were experienced in the human world, it was not possible for the entities to be entirely good and bad at the same time. This natural phenomenon governed by the O-PSO to choose the initial position for the particles in the opposite direction, as well as, in the same direction. Within this theory, the entire swarm was symbolized by the same and opposite particles. The experimental results revealed that proposed O-PSO performed well on many multidimensional dynamic benchmark functions compared to the simple PSO that implemented the uniform distribution for initializing the particles, and the experimental results depicts that O-PSO performed better on several multidimensional complex benchmark functions. Gutiérrez et al. [28] conducted a research of three distinct PSO initialization methods: the opposition-based initialization, the initialization of orthogonal array, and the chaotic initialization.

### 2.2. Artificial Neural Network Training Using PSO

The processing of real-world problem with the initialization of various strategies using the ANN classifier produced a high effect on the performance of the evolutionary algorithms. The classifier with the prearranged initialization techniques was shown to have precision compared to the one using the random distribution.

In [4, 5], optimization of the hidden layer in the neural network was performed. For the optimization process, the author manipulated the uniform distribution-based initialization of feedforward neural networks. Subasi in [29] classified the EMG signals using the uniform random distribution-based PSO along with SVM to diagnose the neuromuscular anarchy. Similarly, the improved swarm optimized functional link artificial neural network (ISO-FLANN) was proposed by Dehuri in [30] using random number initialization following uniform distribution. Optimal Latin Hypercube Design (OLHD) initialization approach was proposed by the authors in [31] and evaluated on several data mining problems with the other quasirandom sequences, such as Faure, Halton, and Sobol sequences. The proposed OLHD was better than quasirandom sequences in terms of efficiency measures.

In [32], the authors introduced the training of NN with particle swarm optimization (NN-PSO) for anticipating the structural failure in reinforced concrete (RC) buildings. The weight vectors for NN was calculated by incorporating PSO on the basis of minimum root mean square error. The introduced NN-PSO classifier was sufficient to handle the structural failure in RC buildings. Xue et al. [33] presented a new strategy for the feedforward neural network (FNN) classifier, in which a self-adaptive parameter and strategy-based PSO (SPS-PSO) was integrated to reduce the dimensions of large-scale optimization problems. A new algorithm by using PSO was proposed in [34], which can spontaneously finalize the most appropriate architecture of deep convolutional neural networks (CNNs) for the classification of images, termed as psoCNN. A novel NN-based training algorithm by incorporating PSO is proposed in [35] called LPSONS. In the LPSONS algorithm, the velocity parameter of PSO was embedded with Mantegna Levy flight distribution for improved diversity. Additionally, the proposed algorithm is used to train feedforward multilayered perceptron ANNs. In [36], PSO was used for feature engineering of diabetic retinopathy, and after it, the NN classifier was applied for the classification of diabetic retinopathy disease.

After conducting a thorough literature review, we can infer that the particle efficiency and convergence velocity are highly dependent on the swarm initialization process. If all the particles with a proper pattern cover the entire search space, there are more chances that the global optimum will be found at an early stage of PSO.

## 3. Particle Swarm Optimization

PSO is a global optimization technique that plays an important role in the fields of applied technology and has been widely deployed in numerous engineering applications, such as preparation of heating systems, data mining, power allocation of cooperative communication networks, pattern recognition, machine learning, optimizing route selection, and information security to name a few. PSO works on the application of candidates. To maximize a problem, the optimal solution is represented by each candidate who is designated as a particle. The current location of the particle is defined by the *n*-dimensional search space and is represented by the vector solution *x*. In the form of a fitness score carried out by particles, each solution is translated. In the *n*-dimensional search space at the *k*th direction, position vector *x* can be calculated by provoking each particle *p.* Velocity vector *v* can be defined as the motion of particles and the step size of an entire swarm in the search space is other than position vector *p*.

PSO begins with the population, consisting of *n* particles that fly at the iteration *k*_*i*_ in the *d*-dimensional search space to look for the optimal solution. Swarm mutation can transform the objective feature into the desired candidate solution. For updating the position and velocity of the particles, the following two equations are used:

(1) $$\begin{array}{c} v_{z+1}=v_{z}+\gamma_{1}\times \left(p_{z}^{\text{best}}-x_{z}\right)+\gamma_{2}\times \left(g_{z}^{\text{best}}-x_{z}\right), \end{array}$$

(2) $$\begin{array}{c} x_{z+1}=x_{z}+v_{z+1}. \end{array}$$

In the above equations, the position vector and velocity vectors are *v*_*z*_ and *x*_*z*_, respectively.*p*_*z*_^best^ shows the local best solution of the entire swarm acquired using its own previous experience, and *g*_*z*_^best^ reflects the global best solution acquired using the *N*-dimension experience of its neighbour. While *γ*_1_ − ⟶*c*1*r*1 and *γ*_2_ − ⟶*c*2*r*2, *c*1 and *c*2 are the acceleration factors that influence the acceleration weights and *r*1 and *r*2 are two random numbers produced by using the random number generator. *x*_*z*+1_ is an updated position vector that guides the novel point at the *k*^th^ iteration for the current particle, where *v*_*z*+1_ is the newly updated velocity. It is possible to drive three different factors from equation (1). The “momentum factor ⟶ *v*_*z*_” represents the old velocity. The “cognitive factor ⟶ *γ*_1_ × (*p*_*z*_^best^ − *x*_*z*_)” gives local best fitness that has taken from all the previous finesses. The “social factor ⟶ *γ*_2_ × (*g*_*z*_^best^ − *x*_*z*_)” provides the best global solution amplified by the intact neighbour particles. The pseudocode of fundamental PSO is present in Algorithm 1.

## 4. Training of the Neural Networks

**Figure 1:**
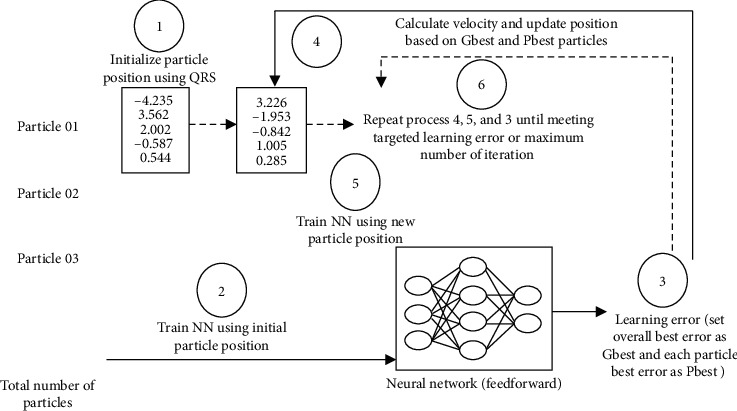
Feedforward neural network.

The artificial neural network (ANN) is perceived as the most effective technique of approximation, which is used to approximate the nonlinear functions and their relationships. The ANN model is capable of generalizing, learning, organizing, and adapting data. The ANN architecture is based on an interlined series of synchronized neurons, whereas the multiprocessing layer is used to compute the encoding of information [37]. ANN is a computational mathematical model that regulates the relationship between the input and output layers of different nonlinear functions [38]. In this study, we have used the feedforward neural network present in Figure 1, which is the most frequently used and popular architecture of the ANN. The feedforward neural network is defined by the three layers, i.e., input layer, sandwich layer, and output layer, respectively. Input layer served as NN gateway, where the information frame is inserted. The intermediate task of the sandwich layer is to execute the data frame using the input layer. The outcomes are derived from the output layer [39]. Both layers' units are connected with the serial layer nodes, and the link between the nodes is structured in the feedforward neural network. Bias is a component of each unit and has a value of −1 as present in [24].

For weight optimization of NN, the position of each particle in swarm shows a set of weight for the current epoch or iteration. The dimensionality of each particle is the number of weights associated with the network. The particle moves within the weight space attempting to minimize learning error (mean squared error (MSE) or sum of squared error (SSE)). In order to change the weights of the neural network, change in Position occurs that will reduce the error in current epoch. There is no backpropagation concept in PSONN where the feedforward NN produced the learning error (particle fitness) based on set of weight and bias (PSO positions).

The challenge of premature convergence is addressed in the problem of weight optimization of ANN [40, 41]. The primary objective of the ANN model is to achieve a set of optimum parameters and weights. The two major classification approaches used to segregate the positive entities from the negative entities are gradient descent and error correction, respectively. Gradient descent-based techniques are low in performance, where the concerns are high dimensional and the parameters are exclusively dependent on the structure. Due to this fact, it stuck in local minima. Backpropagation is one of the gradient decent techniques, which is most commonly used to train the neural network models and solve complex multimodal problems in the real-world as mentioned in [24].

## 5. Random Number Generator

The built-in library function is used to construct the mesh of numbers randomly at uniform locations through Rand (*x*_(min) *x*_max) in [42]. A continuous uniform distribution probability density function describes the effect of uniformity on any sequence. It is possible to characterize the probability density function as given in the following equation:

(3) $$\begin{array}{c} f\left(t\right)=\left\{\begin{array}{c} \frac{1}{p-q},\;\text{for }p<t<q,\\ 0,\;\text{for }t<p\text{ or }t>q, \end{array}\right. \end{array}$$

where *p* and *q* represent the maximum likelihood parameter. Due to the zero impact on the *f* (*t*) d*t* integrals over any length, the value of *f* (*t*) is useless at the boundary of *p* and *q*. The calculation of maximum probability parameter is determined by the estimated probability function, which is given in

(4) $$\begin{array}{c} l\left(p,q|t\right)=n\;\log\left(q-p\right). \end{array}$$

## 6. The Sobol Sequence

The Sobol distribution was undertaken for the reconstruction of coordinates in [43]. The relation of linear recurrences is included for each dimension *d*_*z*_ coordinate, and the binary expression for linear recurrence can be defined for the nonnegative instance *a*_*z*_ as present in

(5) $$\begin{array}{c} a=a_{1}2^{0}+a_{2}2^{1}+a_{3}2^{2}+\cdots +a_{z}2^{z-1}. \end{array}$$

For dimension *d*_*z*_, the instance *i* can be generated using

(6) $$\begin{array}{c} x_{i}^{D}=i_{1}v_{1}^{D}+i_{2}v_{2}^{D}+\cdots +i_{z}v_{z}^{D}. \end{array}$$

*v*_1_^*D*^ denotes the *k* th direction binary function of an instance *v*_*i*_^*D*^ at the dimension *d*_*z*_, and *v*_*i*_^*D*^ can be computed using

(7) $$\begin{array}{c} V_{k}^{D}=c_{1}v_{k-1}^{D}+c_{2}v_{k-2}^{D}+\cdots +c_{z}v_{z-1}^{D}+\left(\frac{v_{i-z}^{D}}{2^{z}}\right), \end{array}$$

where *c*_*z*_ describes polynomial coefficient where *k* *>* *z*.

## 7. The Halton Sequence

In [44], the authors proposed the Halton sequence as an improved variant of the van der Corput sequence. For generating random points, Halton sequences use a coprime base.  Algorithm 2 shows the pseudocode for generating the Halton sequences.

## 8. The WELL Sequence

Panneton et al. [45] suggested the Well Equi-distributed Long-period Linear (WELL) sequence. Initially, it was performed as a modified variant of the Mersenne Twister algorithm. The WELL distribution algorithm is given as in Algorithm 3.

For the WELL distribution, the algorithm mentioned above describes the general recurrence. The algorithm definition is as follows: *x* and *r* are two integers with an interval of *r* > 0 and 0 < *x* < *k* and *k*=*r∗w* − *x*, and *w* is the weight factor of distribution. The binary matrix of size *r∗w* having the *r* bit block is expressed by *A*_0_ to *A*_7_. *m*_*x*_ describes the bitmask that holds the first *w*—*x* bits. *t*_0_ to *t*_7_ are temporary vector variables.

**Figure 2:**
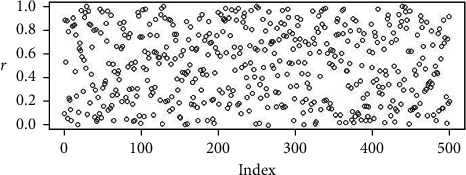
Population initialization using uniform distribution.

**Figure 3:**
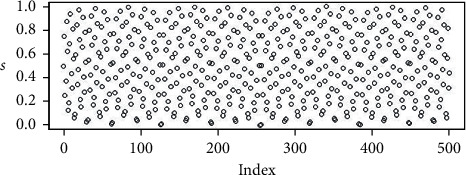
Population initialization using Sobol distribution.

**Figure 4:**
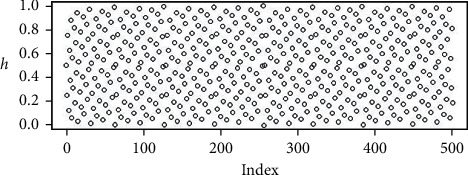
Population initialization using uniform distribution.

**Figure 5:**
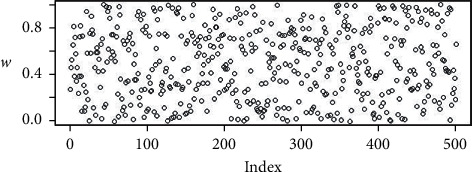
Population initialization using WELL distribution.

The random points in Figures 2–5 are the uniform, and Sobol, Halton, and WELL distributions are represented by the bubble plot in which the *y*-axis is represented by the random values and the *x*-axis is shown in the table by the relevant index of the point concerned.

## 9. Methodology

The objective of this paper is to work out the purity of one of the proposed pseudorandom sequences. Pseudorandom sequences are much more random than quasirandom sequences. PSO is random in nature, so it does not have a specific pattern to guarantee the global optimum solution. Therefore, we have suggested the WELL distribution-based PSO (WE-PSO) by taking advantage of randomness in the PSO. We have compared the WE-PSO with the uniform distribution-based PSO and other quasirandom distributions-based PSO, i.e., Sobol distribution (SO-PSO) and Halton distribution (H-PSO) to ensure the integrity of the proposed approach. Moreover, by training the nine real-world NN problems, we have tested the proposed technique over NN classifiers. The experimental outcomes reflect an unusual improvement over standard PSO with uniform distribution. WE-PSO approach also outperforms SO-PSO and H-PSO approaches as evident in results. Numerical results have shown that the use of WELL distribution to initialize the swarm enhances the efficiency of population-based algorithms in evolutionary computing. In Algorithm 4, the pseudocode for the proposed technique is presented.

## 10. Results and Discussion

WELL-PSO (WE-PSO) technique was simulated in C++ and applied to a computer with the 2.3 GHz Core (*M*) 2 Duo CPU processor specification. A group of fifteen nonlinear benchmark test functions are used to compare the WE-PSO with standard PSO, SO-PSO, and H-PSO for measuring the execution of the WELL-based PSO (WE-PSO) algorithm. Normally, these functions are applied to investigate the performance of any technique. Therefore, we used it to examine the optimization results of WE-PSO in our study. A list of such functions can be found in Table 1. The dimensionality of the problem is seen in Table 1 as *D*, S represents the interval of the variables, and fmin denotes the global optimum minimum value. The simulation parameters are used in the interval [0.9, 0.4] where *c*1 = *c*2 = 1.45, inertia weight *w* is used, and swarm size is 40. The function dimensions are *D* = 10, 20, and 30 for simulation, and a cumulative number of epochs is 3000. All techniques have been applied to similar parameters for comparatively effective results. To check the performance of each technique, all algorithms were tested for 30 runs.

### 10.1. Discussion

The purpose of this study is to observe the unique characteristics of the standard benchmark functions based on the dimensions of the experimental results. Three simulation tests were performed in the experiments, where the following TW-BA characteristics were observed:

- Effect of using different initializing PSO approaches
- Effect of using different dimensions for problems
- A comparative analysis

The objective of this study was to find the most suitable initialization approach for the PSO and to explore WE-PSO with other approaches, such as SO-PSO, H-PSO, and standard PSO during the first experiment. The purpose of the second simulation is to define the essence of the dimension concerning the standard function optimization. Finally, the simulation results of WE-PSO were compared with the standard PSO, SO-PSO, and H-PSO, respectively. Simulation effects have been addressed in depth in the remainder of the article.

The graphical representation of the similarities of WE-PSO with PSO, H-PSO, and SO-PSO is shown in Figures 6to 20. For WE-PSO, we can observe that majority of the estimates have a better convergence curve. The dimensions 10, 20, and 30 of the problem are described in the *x*-axis, while the *y*-axis represents the mean best against each dimension of the problem.

#### 10.1.1. Effect of Using Different Initializing PSO Approaches

In this simulation, PSO is initialized with WELL sequence (WE-PSO) instead of the uniform distribution. The variant WE-PSO is compared with the other initialized approaches including Sobol sequence (SO-PSO), Halton Sequence (H-PSO), and standard PSO. The experimental findings indicate that the higher dimensions are better.

#### 10.1.2. Effect of Using Different Dimensions for Problems

The core objective of this simulation setup is to find the supremacy of the outcomes based on the dimension of the optimization functions. Three dimensions were used for bench mark functions such as *D* = 10, *D* = 20, and *D* = 30 in experiments. In Table 2, the simulation results were presented. From these simulation results, it was observed that the optimization of higher-dimensional functions is more complex, which can be seen from Table 2 where the dimension size is *D* = 20 and *D* = 30.

#### 10.1.3. A Comparative Analysis

WE-PSO is compared to the other approaches, namely, SO-PSO, H-PSO, and the standard PSO, where the true value of each technique with the same nature of the problem is provided for comparison purposes. Table 1 shows the standard benchmark functions and their parameter settings. Table 2 reveals that WE-PSO is better than the standard PSO, SO-PSO, and H-PSO with dimension D-30 and outperforms in convergence. The comparative analysis can be seen from Table 2 in which the standard PSO of the smaller dimension size (*D* = 10, 20) performs well, while the proposed WE-PSO considerably performs well in convergence as the dimension size increases. Hence, WE-PSO is appropriate for higher dimensions. Simulation runs were carried out on HP Compaq with the Intel Core i7-3200 configuration, with a speed of 3.8 GHz with RAM of 6 GB.

**Figure 7:**
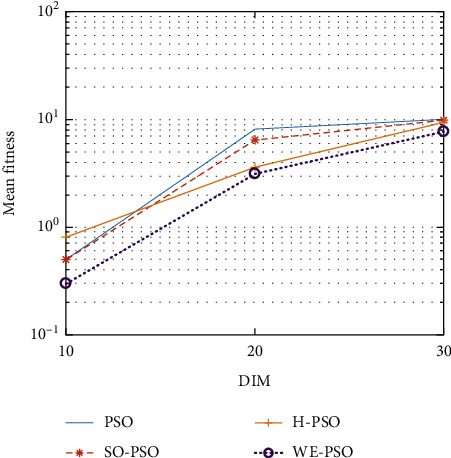
Mean value of function F2.

**Figure 8:**
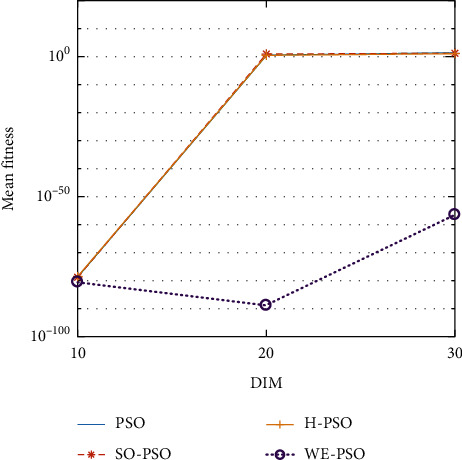
Mean value of function F3.

**Figure 9:**
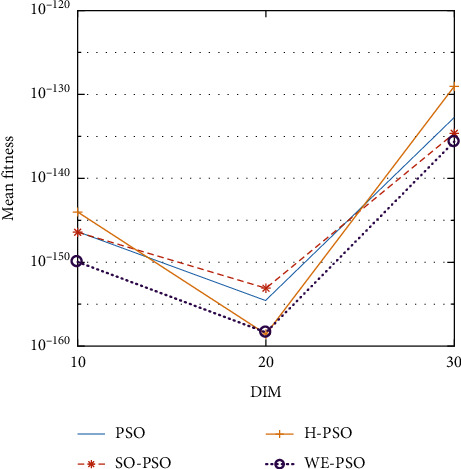
Mean value of function F4.

**Figure 10:**
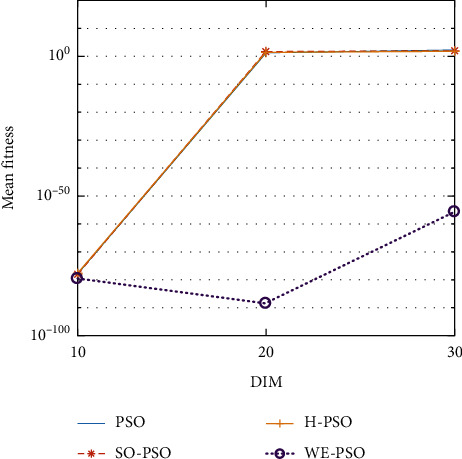
Mean value of function F5.

**Figure 11:**
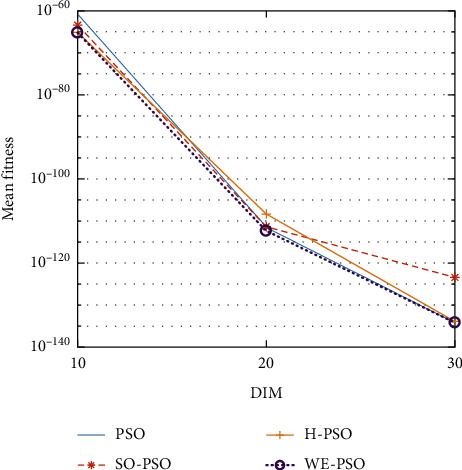
Mean value of function F6.

**Figure 12:**
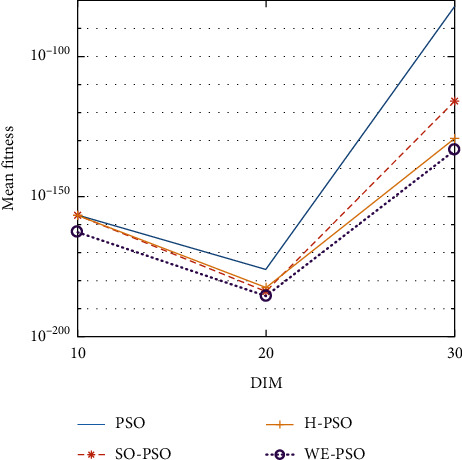
Mean value of function F7.

**Figure 13:**
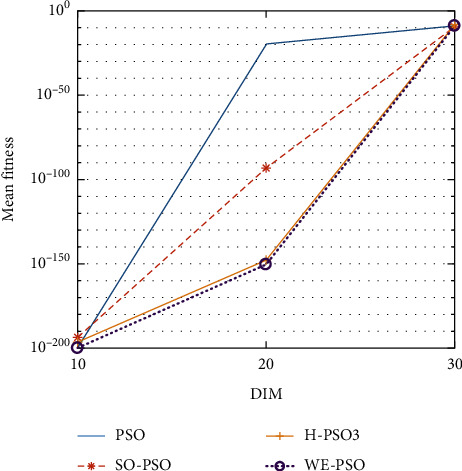
Mean value of function F8.

**Figure 14:**
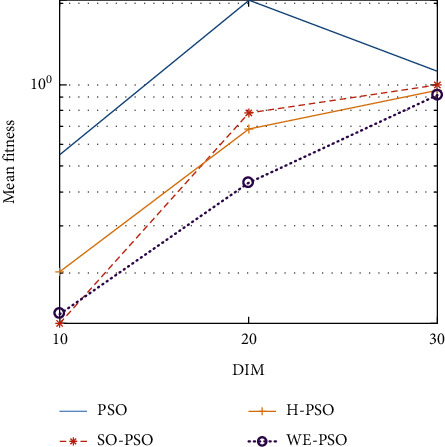
Mean value of function F9.

**Figure 15:**
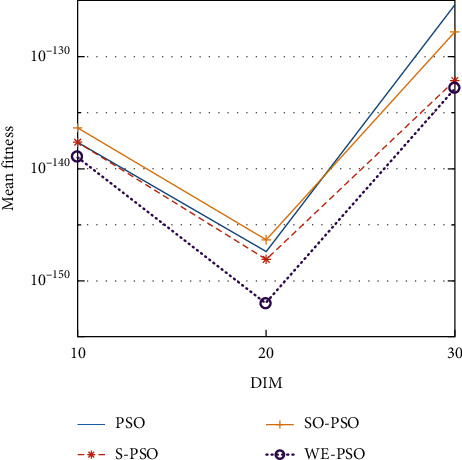
Mean value of function F10.

In contrast with the findings of SO-PSO, H-PSO, and traditional PSO, the experimental results from Table 2 reveal that WE-PSO surpasses the results of the aforementioned variants of PSO. It can be observed that the WE-PSO outperforms in all functions when compared to other techniques, while the other approaches perform as follows: H-PSO performs better on functions F4, F1, and F2 for 20D, but H-PSO gives overall poor results on higher dimensions, and SO-PSO gives slightly better results on the functions F8, F9, and F15 on 10-D but gives worst result on larger dimensions. Figures from Figures 7to 15 depict that WE-PSO outperforms in simulation results than other approaches for solving the dim size *D* = 10, *D* = 20, and *D* = 30 on the standard benchmark test functions.

#### 10.1.4. Statistical Test

To objectively verify the consistency of the findings, the Student *T*-test is performed statistically. For the success of the competing algorithms, the *T* value is computed using

(8) $$\begin{array}{c} t=\frac{\bar{X_{1}}-\bar{X_{2}}}{\sqrt{\left(\left({\text{SD}}_{1}^{2}/\left(n_{1}-1\right)\right)+\left({\text{SD}}_{2}^{2}/\left(n_{2}-1\right)\right)\right)}}. \end{array}$$

*T* value can be positive or negative in the above equation, where $\bar{X_{1}}$ and $\bar{X_{2}}$ reflect the mean value of the first and second samples. The sample size is referred to as *n*_*1*_ and *n*_*2*_ for both samples. The standard deviations for both samples are SD_1_^2^ and SD_2_^2^. Positive and negative values indicate that WE-PSO outperforms other approaches. Student's *T*-test results are presented in Table 3.

## 11. Experiments for Data Classification

A comparative analysis on the real-world benchmark dataset problem is evaluated for the training of neural networks to validate the efficiency of the WE-PSO. Using nine benchmark datasets (Iris, Diabetes, Heart, Wine, Seed, Vertebral, Blood Tissue, Horse, and Mammography) from the world-famous UCI machine-learning repository, we conducted experiments. Training weights are initialized randomly within the interval [−50, 50]. Feedforward neural network accuracy is tested in the form of root mean squared error (RMSE). The features of the datasets that are used can be seen in Table 4.

### 11.1. Discussion

Backpropagation algorithms using standard PSO, SO-PSO, H-PSO, and WE-PSO are trained in the multilayer feedforward neural network. Comparison of these training approaches is tested on real classification datasets that are taken from the UCI repository. The cross-validation method is used to assess the efficiency of various classification techniques. The k-fold cross-validation method is used in this paper for the training of neural networks with the standard PSO, SO-PSO, H-PSO, and proposed algorithm WE-PSO. The k-fold is used with the value *k* = 10 in the experiments. The dataset has been fragmented into 10 chunks; each data chunk comprises the same proportion of each class of dataset. One chunk is used for the testing phase, while nine chunks were used for the training phase. Nine well-known real-world datasets which were taken from UCI were compared with the experimental results of algorithms: standard PSO, SO-PSO, H-PSO, and WE-PSO are used for evaluating the performance. After the simulation, the results showed that the training of neural networks with the WE-PSO algorithm is better in terms of precision and its efficiency is much higher than the traditional approaches. The WE-PSO algorithm can also be used successfully in the future for data classification and statistical problems. The findings of classification accuracy are summarized in Table 5.

## 12. Conclusion

**Figure 21:**
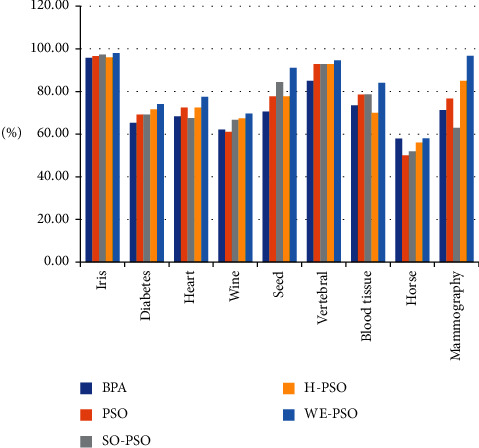
Classification testing accuracy results.

The performance of PSO depends on the initialization of the population. In our work, we have initialized the particles of PSO by using a novel quasirandom sequence called the WELL sequence. However, the velocity and position vector of particles are modified in a random sequence fashion. The importance of initializing the particles by using a quasirandom sequence is highlighted in this study. The experimental results explicitly state that the WELL sequence is optimal for the population initialization, due to its random nature. Moreover, the simulation results have shown that WE-PSO outperforms the PSO, S-PSO and H-PSO approaches. The techniques are also applied to neural network training and provide significantly better results than conventional training algorithms, including standard PSO, S-PSO, and H-PSO approaches, respectively. The solution provides higher diversity and increases the potential to search locally. The experimental results depict that our approach has excellent accuracy of convergence and prevents the local optima. Our technique is much better when it is compared to the traditional PSO and other initialization approaches for PSO as evident in Figure 21. The use of mutation operators with the initialization technique may be evaluated on large-scale search spaces in the future. The core objective of this research is universal but relevant to the other stochastic-based metaheuristic algorithm, which will establish our future direction.

## Figures and Tables

**Figure 6:**
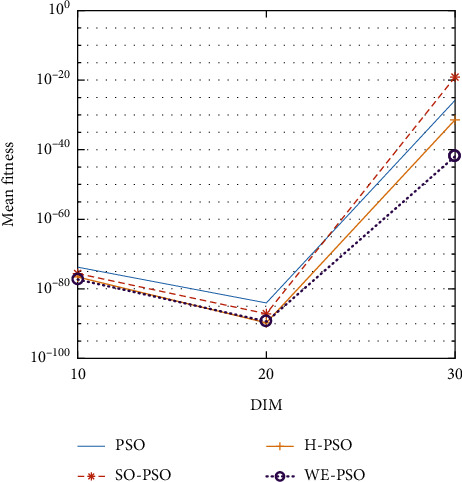
Mean value of function F1.

**Figure 16:**
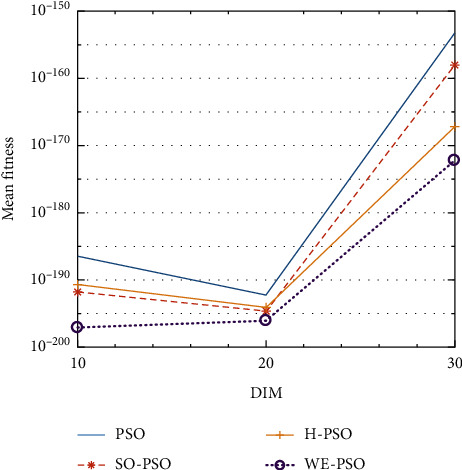
Mean value of function F11.

**Figure 17:**
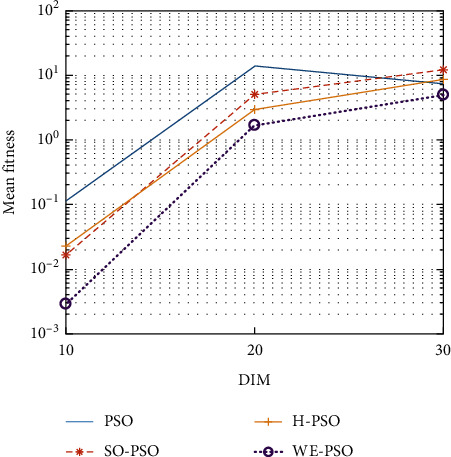
Mean value of function F12.

**Figure 18:**
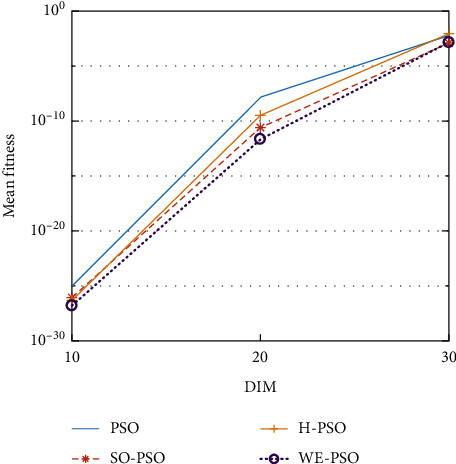
Mean value of function F13.

**Figure 19:**
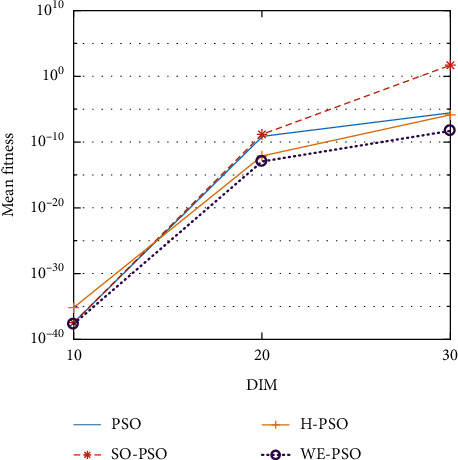
Mean value of function F14.

**Figure 20:**
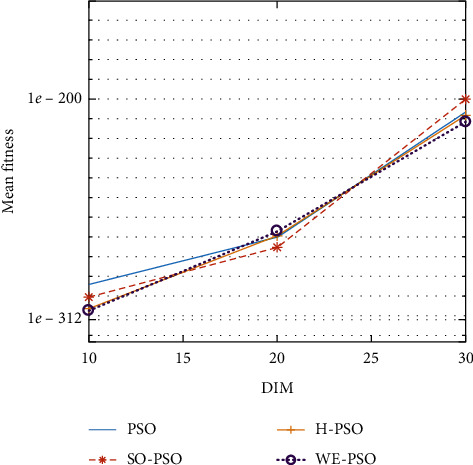
Mean value of function F15.

**Algorithm 1:**
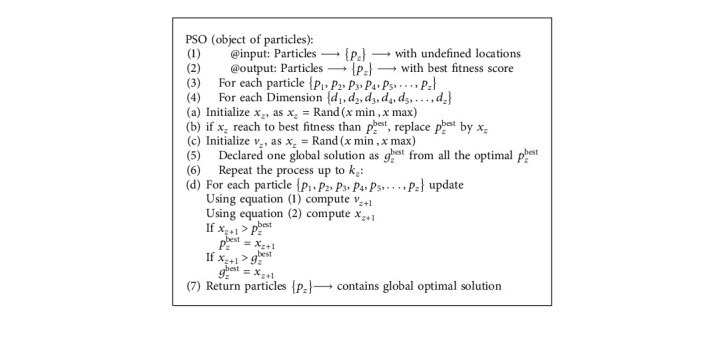
Standard PSO pseudocode.

**Algorithm 2:**
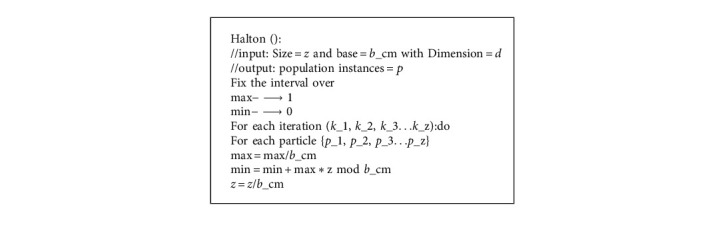
Halton sequences.

**Algorithm 3:**
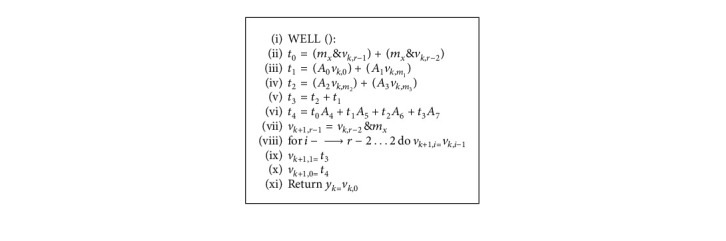
WELL sequences.

**Algorithm 4:**
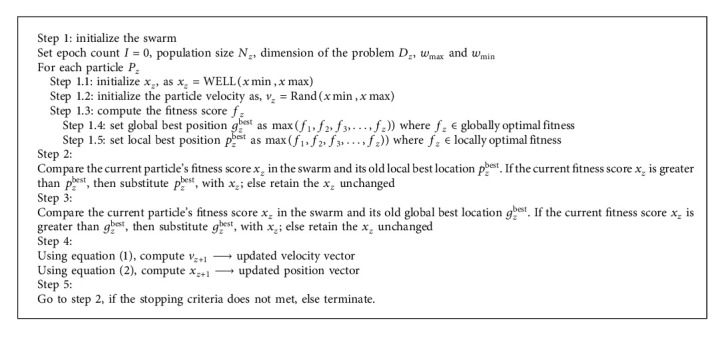
Proposed PSO pseudocode.

**Table 1:** Standard objective functions and their optimal.

SR#	Function name	Objective function	Search space	Optimal value
F1	Sphere	Min *f*(*x*)=∑_*i*=1_^*n*^*x*_*i*_^2^	−5.12 ≤ *x*_*i*_ ≤ 5.12	0
F2	Rastrigin	Min *f*(*x*)=∑_*i*=1_^*n*^[*x*_*i*_^2^ − 10 cos(2*πx*)+10]_*i*_	−5.12 ≤ *x*_*i*_ ≤ 5.12	0
F3	Axis parallel hyper-ellipsoid	Min *f*(*x*)=∑_*i*=1_^*n*^*i* · *x*_*i*_^2^	−5.12 ≤ *x*_*i*_ ≤ 5.12	0
F4	Rotated hyper-ellipsoid	Min *f*(*x*)=∑_*i*_^*n*^(∑_*j*=1_^*i*^*x*_*j*_)^2^	−65.536 ≤ *x*_*i*_ ≤ 65.536	0
F5	Moved axis parallel hyper-ellipsoid	Min *f*(*x*)=∑_*i*=1_^*n*^5*i* · *x*_*i*_^2^	−5.12 ≤ *x*_*i*_ ≤ 5.12	0
F6	Sum of different power	Min *f*(*x*)=∑_*i*=1_^*n*^|*x*_*i*_|^*i*+1^	−1 ≤ *x*_*i*_ ≤ 1	0
F7	Chung Reynolds	Min *f*(*x*)=(∑_*i*=1_^*n*^*x*_*i*_^2^)^2^	−100 ≤ *x*_*i*_ ≤ 100	0
F8	Csendes	Min *f*(*x*)=∑_*i*=1_^*n*^*x*_*i*_^6^(2+sin(1/*x*_*i*_))	−1 ≤ *x*_*i*_ ≤ 1	0
F9	Schaffer	$\text{Min }f\left(x\right)=\sum_{i=1}^{n}0.5+\left(\sin^{2}\sqrt{x_{i}^{2}+x_{i+1}^{2}}-0.5/{\left[1+0.001\left(x_{i}^{2}+x_{i+1}^{2}\right)\right]}^{2}\right)$	−100 ≤ *x*_*i*_ ≤ 100	0
F10	Schumer Steiglitz	Min *f*(*x*)=∑_*i*=1_^*n*^*x*_*i*_^4^	−5.12 ≤ *x*_*i*_ ≤ 5.12	0
F11	Schwefel	Min *f*(*x*)=∑_*i*=1_^*n*^*x*_*i*_^*α*^	−100 ≤ *x*_*i*_ ≤ 100	0
F12	Schwefel 1.2	Min *f*(*x*)=∑_*i*_^*D*^(∑_*j*=1_^*i*^*x*_*j*_)^2^	−100 ≤ *x*_*i*_ ≤ 100	0
F13	Schwefel 2.21	Min *f*(*x*)=max|*x*_*i*_|_1<*i*<*D*_	−100 ≤ *x*_*i*_ ≤ 100	0
F14	Schwefel 2.22	Min *f*(*x*)=∑_*i*=1_^*D*^|*x*_*i*_|+∏_*i*=1_^*n*^|*x*_*i*_|	−100 ≤ *x*_*i*_ ≤ 100	0
F15	Schwefel 2.23	Min *f*(*x*)=∑_*i*=1_^*n*^*x*_*i*_^10^	−10 ≤ *x*_*i*_ ≤ 10	0

**Table 2:** Comparative results among the four PSO algorithms on 15 benchmark test functions.

F#	Iter	DIM	PSO		SO-PSO		H-PSO		WE-PSO	
			Mean	Std. dev	Mean	Std. dev	Mean	Std. dev	Mean	Std. dev
1	1000	10	2.33*E* − 74	7.36*E* − 74	2.74*E* − 76	8.66*E* − 76	3.10*E* − 77	9.79*E* − 77	**5.91*E*** − **78**	**1.87*E*** − **77**
	2000	20	1.02*E* − 84	3.22*E* − 84	8.20*E* − 88	2.59*E* − 87	**1.76*E*** − **90**	**5.58*E*** − **90**	4.95*E* − 90	1.48*E* − 89
	3000	30	1.77*E* − 26	5.32*E*− 26	7.67*E* − 20	2.30*E* − 19	4.13*E* − 32	1.24*E* − 31	**1.30*E*** − **42**	**3.90*E*** − **42**

2	1000	10	4.97*E* − 01	1.49*E* + 00	4.97*E* − 01	1.49*E* + 00	7.96*E* − 01	2.39*E* + 00	**2.98*E*** − **01**	**8.95*E*** − **01**
	2000	20	8.17*E* + 00	2.29*E* + 01	6.47*E* + 00	1.91*E* + 01	3.58*E* + 00	**9.79*E*** **+** **00**	**3.11*E*** **+** **00**	1.10*E* + 01
	3000	30	1.01*E* + 01	2.95*E* + 01	9.86*E* + 00	2.76*E* + 01	9.45*E* + 00	27.6991	**7.76*E*** **+** **00**	**2.20*E*** **+** **01**

3	1000	10	8.70*E* − 80	2.61*E* − 79	1.79*E* − 79	5.37*E* − 79	4.87*E* − 79	1.46*E* − 78	**4.40*E*** − **81**	**1.32*E*** − **80**
	2000	20	2.62144	7.86*E* + 00	7.86432	2.36*E* + 01	2.62144	7.86*E* + 00	**1.78*E*** − **89**	**5.33*E*** − **89**
	3000	30	2.62*E* + 01	7.86*E* + 01	1.57*E* + 01	4.72*E* + 01	1.05*E* + 01	31.4573	**3.87*E*** − **57**	**1.16*E*** − **56**

4	1000	10	4.46*E* − 147	1.34*E* − 146	3.86*E* − 147	1.16*E* − 146	9.78*E* − 145	2.93*E* − 144	**1.24*E*** − **150**	**3.73*E*** − **150**
	2000	20	3.14*E* − 155	9.41*E* − 155	9.27*E* − 154	2.78*E* − 153	**2.75*E*** − **159**	**8.24*E*** − **159**	4.96*E* − 159	1.49*E* − 158
	3000	30	1.82*E* − 133	5.45*E* − 133	2.36*E* − 135	7.09*E* − 135	8.53*E* − 130	2.56*E* − 129	**2.54*E*** − **136**	**7.62*E*** − **136**

5	1000	10	4.35*E* − 79	1.30*E* − 78	8.95*E* − 79	2.69*E* − 78	2.43*E* − 78	7.30*E* − 78	**2.20*E*** − **80**	**6.61*E*** − **80**
	2000	20	1.31*E* + 01	3.93*E* + 01	3.93*E* + 01	1.18*E* + 02	1.31*E* + 01	3.93*E* + 01	**3.12*E*** − **89**	**9.36*E*** − **89**
	3000	30	1.31*E* + 02	3.93*E* + 02	7.86*E* + 01	2.36*E* + 02	5.24*E* + 01	1.57*E* + 02	**1.94*E*** − **56**	**5.81*E*** − **56**

6	1000	10	1.70*E* − 61	5.11*E* − 61	4.45*E* − 64	1.33*E* − 63	7.29*E* − 66	2.19*E* − 65	**4.62*E*** − **66**	**1.39*E***− **65**
	2000	20	3.25*E* − 112	9.74*E* − 112	4.39*E* − 112	1.32*E* − 111	5.01*E* − 109	1.50*E* − 108	**4.45*E*** − **113**	**1.34*E*** − **112**
	3000	30	7.21*E* − 135	2.16*E* − 134	4.10*E* − 124	1.23*E* − 123	1.51*E* − 134	4.54*E* − 134	**6.96*E*** − **135**	**2.09*E*** − **134**

7	1000	10	2.96*E* − 157	8.87*E* − 157	2.39*E* − 157	7.18*E* − 157	1.28*E* − 157	3.84*E* − 157	**2.47*E*** − **163**	0.00*E* + 00
	2000	20	8.79*E* − 177	0.00*E* + 00	1.77*E* − 184	0.00*E* + 00	3.49*E* − 183	0.00*E* +00	**3.41*E*** −**186**	0.00*E* + 00
	3000	30	1.23*E* − 82	3.68*E*v− 82	1.25*E* − 116	3.74*E* − 116	5.99*E* − 130	5.99*E* − 130	**4.60*E*** − **134**	**1.38*E*** − **133**

8	1000	10	4.39*E* − 200	0.00*E* + 00	1.98*E* − 194	0.00*E* + 00	4.51*E* − 197	0.00*E* + 00	**8.99*E*** − **201**	**0.00*E*** **+** **00**
	2000	20	1.57*E* − 20	4.70*E* − 20	1.04*E* − 93	3.13*E* − 93	1.10*E* − 148	3.30*E* − 148	**4.09*E*** − **151**	**1.23*E*** − **150**
	3000	30	1.89*E* − 09	5.68*E* − 09	**4.54*E*** − **10**	**1.36*E*** − **09**	1.14*E* − 08	3.43*E* − 08	1.34*E* − 09	4.03E − 09

9	1000	10	5.49*E* − 01	6.72*E* − 01	**1.30*E*** − **01**	2.02*E* − 01	2.02*E* −01	5.73*E* − 01	1.42*E* − 01	**1.42*E*** − **01**
	2000	20	2.05*E* + 00	1.31*E* + 00	7.83*E* − 01	1.43*E* + 00	6.83*E* − 01	1.29*E* + 00	**4.32*E*** − **01**	**1.08*E*** **+** **00**
	3000	30	1.12*E* + 00	2.39*E* + 00	9.99*E* − 01	2.30*E* + 00	9.56*E* − 01	2.52*E* + 00	**9.12*E*** − **01**	**2.23*E*** **+** **00**

10	1000	10	2.23*E* − 138	2.23*E* − 138	2.23*E* − 138	3.15*E* − 137	4.35*E* − 137	1.31*E* − 136	**1.10*E*** − **139**	**3.31*E*** − **139**
	2000	20	3.79*E* − 148	1.14*E* − 147	7.87*E* − 149	2.36*E* − 148	4.19*E* − 147	1.26*E* − 146	**8.73*E*** − **153**	**2.62*E*** − **152**
	3000	30	4.43*E* − 126	1.33*E* − 125	7.52*E* − 133	2.26*E* − 132	1.57*E* − 128	4.71*E* − 128	**1.38*E*** − **133**	**4.14*E*** − **133**

11	1000	10	3.75*E* − 187	0.00*E* + 00	1.57*E* − 192	0.00*E* + 00	2.15*E* − 191	0.00*E* + 00	**8.99*E*** − **198**	0.00*E* + 00
	2000	20	5.29*E* − 193	0.00*E* + 00	2.53*E* − 195	0.00*E* + 00	8.45*E* − 195	0.00*E* + 00	**9.83*E*** − **197**	0.00*E* + 00
	3000	30	4.82*E* − 154	1.44*E* − 153	8.84*E* − 159	2.65*E* − 158	5.49*E* − 168	0.00*E* + 00	**5.75*E*** −**173**	0.00*E* + 00

12	1000	10	1.13*E* − 01	3.40*E* − 01	1.67*E* − 02	5.02*E* − 02	2.28*E* − 02	6.85*E* − 02	**2.89*E*** − **03**	**8.66*E*** − **03**
	2000	20	1.39*E* + 01	4.12*E* + 01	5.03*E* + 00	1.50*E* + 01	2.95*E* + 00	8.84*E* + 00	**1.67*E*** **+** **00**	**5.01*E*** **+** **00**
	3000	30	7.45*E* + 00	2.23*E* + 01	1.22*E* + 01	3.66*E* + 01	8.74*E* + 00	2.60*E* + 01	**4.94*E*** **+** **00**	**1.48*E*** **+** **01**

13	1000	10	8.04*E* − 26	2.41*E* − 25	8.01*E* − 27	2.40*E* − 26	3.59*E* − 27	1.08*E* − 26	**1.41*E*** − **27**	**1.02*E*** − **26**
	2000	20	1.42*E* − 08	4.26*E* − 08	2.64*E* − 11	7.93*E* − 11	3.29*E* − 10	9.86*E* − 10	**2.14*E*** − **12**	**6.43*E*** − **12**
	3000	30	6.20*E* − 03	1.86*E* − 02	1.41*E* − 03	4.23*E* − 03	9.36*E* − 03	2.81*E* − 02	**1.41*E*** − **03**	**3.83*E*** − **03**

14	1000	10	3.62*E* − 38	1.09*E* − 37	3.62*E* − 38	1.09*E* − 37	5.92*E* − 36	1.77*E* − 35	**1.95*E*** − **38**	**5.86*E*** − **38**
	2000	20	6.27*E* − 10	1.88*E* − 09	1.38*E* − 09	4.14*E* − 09	7.91*E* − 13	2.37*E* − 12	**1.17*E*** −**13**	**3.51*E*** − **13**
	3000	30	2.56*E* − 06	7.67*E* − 06	4.80*E* + 01	1.44*E* + 02	1.34*E* − 06	4.03*E* − 06	**4.88*E*** − **09**	**1.46*E*** − **08**

15	1000	10	1.10*E* − 294	0.00*E* + 00	3.19*E* − 301	0.00*E* + 00	2.78*E* − 307	0.00*E* + 00	**3.21*E*** − **308**	0.00*E* + 00
	2000	20	6.16*E* − 271	0.00*E* + 00	**5.09*E*** − **276**	0.00*E* + 00	3.74*E* − 270	0.00*E* + 00	4.85*E* − 268	0.00*E* + 00
	3000	30	3.08*E* − 207	0.00*E* + 00	1.04*E* − 200	0.00*E* + 00	8.12*E* − 209	0.00*E* + 00	**3.06*E*** − **212**	0.00*E* + 00

Note: “‘Mean”' shows mean value and “Std. dev” indicates the standard deviation. The best results among the four PSO algorithms are presented in bold.

**Table 3:** Results of Student's *T*-test for all techniques.

F#	Iter	DIM	WE-PSO vs. PSO		WE-PSO vs. SO-PSO		WE-PSO vs. H-PSO	
			*T*-value	Sig	*T*-value	Sig	*T*-value	Sig
1	1000	10	**+1.02**	WE-PSO	**+0.99**	WE-PSO	**+0.75**	WE-PSO
	2000	20	**+1.00**	WE-PSO	**+0.48**	WE-PSO	−0.83	H-PSO
	3000	30	**+1.00**	WE-PSO	**+1.00**	WE-PSO	**+1.00**	WE-PSO

2	1000	10	**+30.71**	WE-PSO	**+15.67**	WE-PSO	**+1.21**	WE-PSO
	2000	20	**+8.82**	WE-PSO	**+107.56**	WE-PSO	**+11.13**	WE-PSO
	3000	30	**+0.63**	WE-PSO	**+34.29**	WE-PSO	**+0.65**	WE-PSO

3	1000	10	**+099**	WE-PSO	**+1.00**	WE-PSO	**+0.83**	WE-PSO
	2000	20	**+1.00**	WE-PSO	**+1.00**	WE-PSO	**+263.14**	WE-PSO
	3000	30	**+1.00**	WE-PSO	**+525.29**	WE-PSO	**+0.93**	WE-PSO

4	1000	10	**+0.19**	WE-PSO	**+0.99**	WE-PSO	**+1.00**	WE-PSO
	2000	20	**+0.99**	WE-PSO	**+0.84**	WE-PSO	−0.98	H-PSO
	3000	30	**+0.86**	WE-PSO	**+0.26**	WE-PSO	**+0.97**	WE-PSO

5	1000	10	**+0.79**	WE-PSO	**+0.44**	WE-PSO	**+0.98**	WE-PSO
	2000	20	**+0.29**	WE-PSO	**+0.57**	WE-PSO	**+263.14**	WE-PSO
	3000	30	**+0.06**	WE-PSO	**+2622.44**	WE-PSO	**+0.96**	WE-PSO

6	1000	10	**+0.80**	WE-PSO	**+0.98**	WE-PSO	**+0.17**	WE-PSO
	2000	20	**+0.86**	WE-PSO	**+0.96**	WE-PSO	**+0.96**	WE-PSO
	3000	30	**+0.99**	WE-PSO	**+0.98**	WE-PSO	**+0.89**	WE-PSO

7	1000	10	**+0.90**	WE-PSO	**+0.95**	WE-PSO	**+1.00**	WE-PSO
	2000	20	**+1.00**	WE-PSO	**+1.00**	WE-PSO	**+1.00**	WE-PSO
	3000	30	**+1.00**	WE-PSO	**+1.00**	WE-PSO	**+1.00**	WE-PSO

8	1000	10	**+0.75**	WE-PSO	**+0.98**	WE-PSO	**+0.55**	WE-PSO
	2000	20	**+483.97**	WE-PSO	**+1.00**	WE-PSO	**+0.91**	WE-PSO
	3000	30	**+1.41**	WE-PSO	−6.89	SO-PSO	**+522.24**	WE-PSO

9	1000	10	**+53.67**	WE-PSO	−3.00	SO-PSO	**+82.30**	WE-PSO
	2000	20	**+84.84**	WE-PSO	**+33.46**	WE-PSO	**+16.08**	WE-PSO
	3000	30	**+470.01**	WE-PSO	**+390.54**	WE-PSO	**+416.26**	WE-PSO

10	1000	10	**+1.00**	WE-PSO	**+0.84**	WE-PSO	**+0.67**	WE-PSO
	2000	20	**+1.00**	WE-PSO	**+0.81**	WE-PSO	**+0.89**	WE-PSO
	3000	30	**+1.00**	WE-PSO	**+0.98**	WE-PSO	**+0.95**	WE-PSO

11	1000	10	**+0.97**	WE-PSO	**+1.92**	WE-PSO	**+1.00**	WE-PSO
	2000	20	**+1.00**	WE-PSO	**+1.00**	WE-PSO	**+1.00**	WE-PSO
	3000	30	**+0.87**	WE-PSO	**+0.98**	WE-PSO	**+1.00**	WE-PSO

12	1000	10	**+0.91**	WE-PSO	**+0.58**	WE-PSO	**+0.27**	WE-PSO
	2000	20	**+2.26**	WE-PSO	**+1.08**	WE-PSO	**+0.27**	WE-PSO
	3000	30	**+1.84**	WE-PSO	**+2.25**	WE-PSO	**+2.41**	WE-PSO

13	1000	10	**+0.98**	WE-PSO	**+0.48**	WE-PSO	**+0.84**	WE-PSO
	2000	20	**+0.72**	WE-PSO	**+0.78**	WE-PSO	**+0.98**	WE-PSO
	3000	30	**+0.11**	WE-PSO	**+0.39**	WE-PSO	**+0.86**	WE-PSO

14	1000	10	**+0.57**	WE-PSO	**+0.15**	WE-PSO	**+0.82**	WE-PSO
	2000	20	**+0.151**	WE-PSO	**+1.49**	WE-PSO	**+1.50**	WE-PSO
	3000	30	**+0.90**	WE-PSO	**+1.32**	WE-PSO	**+1.32**	WE-PSO

15	1000	10	**+1.00**	WE-PSO	**+1.00**	WE-PSO	**+1.00**	WE-PSO
	2000	20	**+1.00**	WE-PSO	−0.50	SO-PSO	**+0.99**	WE-PSO
	3000	30	**+0.83**	WE-PSO	**+1.00**	WE-PSO	**+1.00**	WE-PSO

**Table 4:** Dataset description.

S. no.	Datasets	Number of total units	Disc feature	Nature	No. of inputs	No. of classes
1	Iris	150	—	Real	4	3
2	Diabetes	768	—	Real	8	2
3	Heart	270	—	Real	13	2
4	Wine	178	—	Real	13	3
5	Seed	210	—	Real	7	3
6	Vertebral	310	—	Real	6	2
7	Blood tissue	748	—	Real	5	2
8	Horse	368	—	Real	27	2
9	Mammography	961	—	Real	6	2

**Table 5:** Classification accuracy results.

S. no.	Datasets	Type	BPA		PSO		SO-PSO		H-PSO		WE-PSO	
			Tr. acc (%)	Ts. acc (%)	Tr. acc (%)	Ts. acc (%)	Tr. acc (%)	Ts. acc (%)	Tr. acc (%)	Ts. acc (%)	Tr. acc (%)	Ts. acc (%)
1	Iris	3-Class	98.2	95.7	99	96.6	98.8	97.3	98.9	96	99.2	**98**
2	Diabetes	2-Class	86.1	65.3	88.7	69.1	89.3	69.1	88.4	71.6	90.4	**74.1**
3	Heart	2-Class	78.5	68.3	99.5	72.5	99.13	67.5	99.13	72.5	100	**77.5**
4	Wine	3-Class	67.3	62.17	74.24	61.11	81.81	66.66	75.75	67.44	75.75	**69.6**
5	Seed	3-Class	84.2	70.56	97.57	77.77	87.27	84.44	98.18	77.77	98.18	**91.11**
6	Vertebral	2-Class	91.4	84.95	96.03	92.85	96.42	92.85	96.40	92.85	97.61	**94.64**
7	Blood tissue	2-Class	76.3	73.47	90.8	78.6	86.94	78.66	83.89	70	84.74	**84**
8	Horse	2-Class	64.4	57.87	69.02	50	74.19	52	72.90	56	**79.35**	**58**
9	Mammography	2-Class	77.36	71.26	80.82	76.66	68.94	63	88	85	97.71	**96.66**

## Data Availability

The data used to support the findings of this study are available from the corresponding author upon reasonable request.
